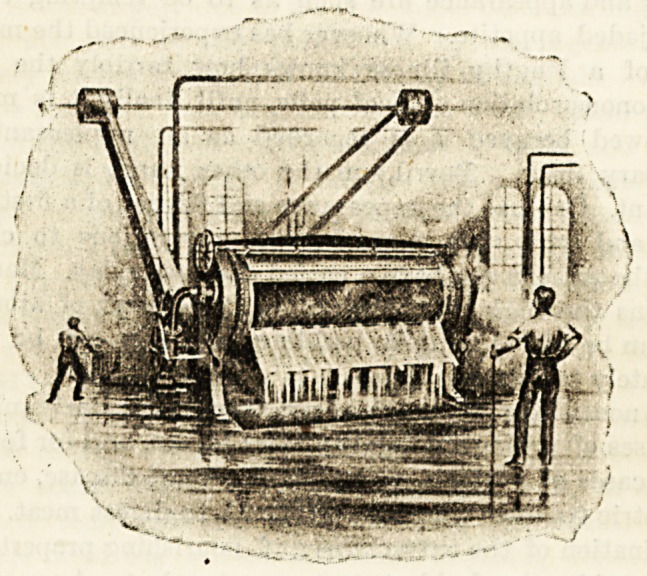# Practical Departments

**Published:** 1902-02-08

**Authors:** 


### PRACTICAL DEPARTMENTS.

### AN ECONOMICAL WASHING-MACHINE.

(Messrs. ISAAC BRAITHWAITE & SON, Kendal, and 24 Garlick Hill, London, E.C.).

A WASHING-MACHINE that promises not to wear or tear the clothes is one that is sure of its welcome and this is the distinctive feature of the "Ibis," produced by Messrs. Braithwaite and Son. The principle in all washing-machines is first to soften and loosen the dirt by a preparation of hot soap, soda, or both. But whereas in the majority of machines the dirt thus loosened is removed by violently dashing the clothes against each other and against the sides of the cylinder, in the "Ibis" they are turned gently over and over, every part being thus exposed to strong jets of soap solution, kept in motion by a powerful centrifugal pump. The cylinder is smaller than in an ordinary machine, being about half the diameter in size, and only nine or ten revolutions are made per minute, instead of 25 or 30. This means that during the whole operation of washing, any point on the circumference of the "Ibis" cylinder travels about 3,250 feet, whereas in the ordinary machines the distance travelled is 20,000 feet. This represents the difference in the wear and tear of the clothes. Then, again, the clothes are not "roped" together, but suitably distributed in the cylinder, and when the process is finished they are dropped out all at once; the opening is large enough for this to be done instantaneously and without the necessity of pulling or forcing in any way. There are other advantages, but this, of saving what a foreigner has called the "abuse" of the clothes during the process of cleansing, is the one that will appeal forcibly to those who have suffered much at the hands of the laundress, whether as individuals or as trustees or officials of public institutions. The details of construction are as follows:—The machine consists essentially of four parts: The casing or enclosing cylinder; the washing or inner cylinder into which the clothes are put; a trough or reservoir in two compartments; a centrifugal circulating pump. The casing is made of sheet iron and surrounds the washing cylinder to protect it, and also to receive the washing and rinsing liquids as they come from the washing cylinder, and to return them to the reservoir. The casing is furnished with a large opening corresponding with the opening in the washing cylinder, which opening can be turned by means of spur gear and a hand wheel to the required position for charging, discharging, or closing the machine. The closing is effected by turning the opening to the top and dropping the lid, which can readily be raised and lowered. The washing cylinder is made of heavy iron plate perforated and thoroughly galvanised; around and lengthwise of its inner circumference, are a number of tubes with small perforations for jets, which all face to the centre of the cylinder. These tubes are connected with a chamber at one end of the cylinder (formed by having a double end), and this chamber is in communication, through the trunnion and stuffing-box, with the centrifugal pump, which delivers the washing liquor at considerable pressure and forces it through the perforated tube on to the clothes in the cylinder. During the washing process, the cylinder, is very slowly rotated, first in one direction, then in the other, by means of the rack and pinion gear. The reservoir consists of a cast-iron vessel divided into two compartments; the one for the soap solution, the other for the rinsing, blueing, or starching water. Below the reservoir runs a large suction channel to the pump, communicating with each of the compartments through a large valve, which can be instantly opened or closed when changing from the soaping to the rinsing process.

The "Ibis" is not a cheap machine as far as initial expense is concerned, but it appears to be one that will prove truly economical in the long run. It obtained the Grand Prix at the Paris Exhibition in 1900, and is made in two sizes.

## Figures and Tables

**Figure f1:**